# Changes Over Time in the Oregon Physician Orders for Life-Sustaining Treatment Registry: A Study of Two Decedent Cohorts

**DOI:** 10.1089/jpm.2018.0446

**Published:** 2019-05-07

**Authors:** Dana M. Zive, Valerie M. Jimenez, Erik K. Fromme, Susan W. Tolle

**Affiliations:** ^1^ Center for Policy and Research in Emergency Medicine, Oregon Health & Science University, Portland, Oregon.; ^2^ Center for Ethics in Health Care, Oregon Health & Science University, Portland, Oregon.; ^3^ Department of Psychosocial Oncology and Palliative Care, Dana-Farber Cancer Institute, Boston, Massachusetts.; ^4^ Division of General Internal Medicine and Geriatrics, Center for Ethics in Health Care, Oregon Health & Science University, Portland, Oregon.

**Keywords:** advance care planning, frailty, POLST, resuscitation orders, serious illness

## Abstract

***Background:*** The Physician Orders for Life-Sustaining Treatment (POLST) began in Oregon in 1993 and has since spread nationally and internationally.

***Objectives:*** Describe and compare demographics and POLST orders in two decedent cohorts: deaths in 2010–2011 (Cohort 1) and in 2015–2016 (Cohort 2).

***Design:*** Descriptive retrospective study.

***Setting/Subjects:*** Oregon decedents with an active form in the Oregon POLST Registry.

***Measurements:*** Oregon death records were matched with POLST orders. Descriptive analysis and logistic regression models assess differences between the cohorts.

***Results:*** The proportion of Oregon decedents with a registered POLST increased by 46.6% from 30.9% (17,902/58,000) in Cohort 1 to 45.3% (29,694/65,458) in Cohort 2. The largest increase (83.3%) was seen in decedents 95 years or older with a corresponding 78.7% increase in those with Alzheimer's disease and dementia, while the interval between POLST form completion and death in these decedents increased from a median of 9–52 weeks. Although orders for do not resuscitate and other orders to limit treatment remained the most prevalent in both cohorts, logistic regression models confirm a nearly twofold increase in odds for cardiopulmonary resuscitation and full treatment orders in Cohort 2 when controlling for age, sex, race, education, and cause of death.

***Conclusion:*** Compared with Cohort 1, Cohort 2 reflected several trends: a 46.6% increase in POLST Registry utilization most marked in the oldest old, substantial increases in time from POLST completion to death, and disproportionate increases in orders for more aggressive life-sustaining treatment. Based on these findings, we recommend testing new criteria for POLST completion in frail elders.

## Introduction

In an era of increased awareness, training, and implementation of advance care planning (ACP), a population-based approach that offers every patient the opportunity to participate is both valuable and within reach.^1^ The challenges and complexities inherent in any such approach create both benefit and risk, and so, it is imperative to have data systems that support safety and improvement. The Physician Orders for Life-Sustaining Treatment (POLST) began in Oregon in 1993 and has since spread widely nationally and internationally.^2–5^ POLST-like forms allow patient preferences to be translated into actionable life-sustaining treatment orders that are portable across settings of care.^3–19^

To ensure that POLST forms were accessible in an emergency, in December 2009, an Oregon statute required that health professionals submit completed POLST forms to a statewide electronic registry (the Oregon POLST Registry) unless a patient chose to opt out.^20^ Since then, the Registry has received over 350,000 forms for over 220,000 patients and submissions to the Registry have steadily increased from 39,875 in 2010 to 56,950 in 2016.^21^ We have previously reported data from the first years of Registry operations characterizing its demographics, life-sustaining treatment order prevalence, and timing of submission in relation to death. It was understood at the time that these data would likely change over time as the Registry became more established and “mature.”^15^ Therefore, with the Registry's continued growth, we sought to re-examine these metrics to assess the growth, change, and implications of Registry maturation in the context of the changing health care environment.

## Methods

The Oregon Health & Science University and the Oregon Public Health Department Institutional Review Boards deemed the study exempt because all data pertained to deceased persons.

**FIG. 1.**
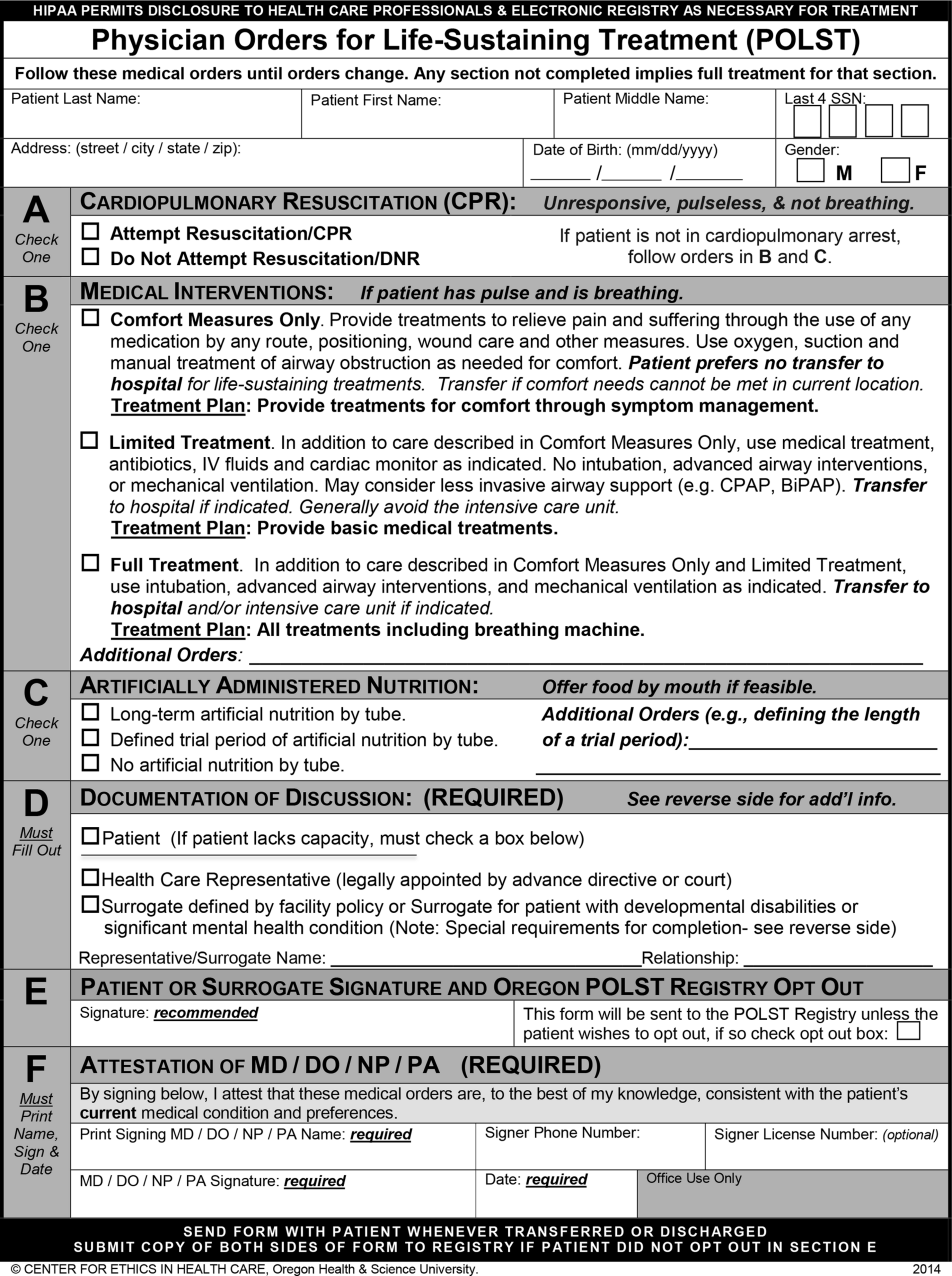
The 2014 Oregon POLST form. POLST, Physician Orders for Life-Sustaining Treatment.

The population included in the sample was Oregonians who died of natural causes in 2010 and 2011 (Cohort 1) and in 2015 and 2016 (Cohort 2). Death certificate data were provided from the Oregon Center for Health Statistics and included date of death, primary cause of death, age at death, location of death, decedent residence information, decedent date of birth, decedent race and ethnicity, and decedent educational attainment. POLST data were captured from the Oregon POLST Registry, matched deterministically with the death records, followed by manual review. Only the form completed closest to death for each registrant was included for those with multiple forms. POLST Registry data included final form orders for Section A: cardiopulmonary resuscitation (CPR) versus do not resuscitate (DNR) (attempt cardiopulmonary resuscitation vs. do not attempt resuscitation) and Section B: medical interventions (comfort measures only [CMO], limited treatment, or full treatment), as well as date of signature (Fig. 1).

### Analysis

Univariate (*t*-test and chi-square) and multivariate analyses were used to assess associations between demographic variables, having a POLST, POLST orders, and cohort. Variables from these unadjusted analyses showing significant differences were included in the logistic regression models. The models examined whether increases in the proportion of orders for life-sustaining treatment, particularly orders for “attempt resuscitation” and “full treatment,” could be attributed to changes in demographics and diagnosis. Two primary models were developed; the dependent variable in the first model was having a POLST order of “attempt resuscitation” and the second model was having a final POLST order of “full treatment,” each stratified by cohort. Analyses were conducted using IBM SPSS Statistics v. 25 (IBM Corp, Armonk, NY).

## Results

The total number of deaths from natural causes increased in Oregon by 7498 (12.9%) from 58,000 to 65,458 between time periods, while the number of forms increased by a much greater magnitude: 11,792 (65.9%) from 17,902 to 29,694 (Table 1). Thus, the proportion of Oregon decedents with a registered POLST when they died increased by 46.6% from 30.9% in Cohort 1 to 45.3% in Cohort 2 (Table 2). This 46.6% proportional increase in POLST use was not seen equally across all groups.

**Table 1.** Demographics of Two Oregon Decedent Cohorts (2010–2011 and 2015–2016) with Proportion of Registered Physician Orders for Life-Sustaining Treatment Forms at Death and Proportional Change between Cohorts

	*Cohort 1: 2010–2011 decedents*			*Cohort 2: 2015–2016 decedents*			
	*Registered POLST (*n* = 17,902)* n	*All natural deaths (*n* = 58,000)* n	*Registered POLST (30.9% of deaths)%*	*Registered POLST (*n* = 29,694)* n	*All natural deaths (*n* = 65,498)* n	*Registered POLST (45.3% of deaths)%*	*Difference Change in % between cohorts*
Age							
≤18	32	536	6.0	33	492	6.7	11.7
19–44	212	1271	16.7	246	1450	17.0	1.8
45–64	2473	10,454	23.7	3185	11,328	28.1	18.6
65–74	2850	9717	29.3	5064	13,118	38.6	31.7
75–84	5068	14,953	33.9	7726	15,867	48.7	43.7
85–94	6083	17,416	34.9	10,676	18,587	57.4	64.5
≥95	1184	3653	32.4	2764	4656	59.4	83.3
Sex							
Female	9739	29,860	32.6	16,421	33,016	49.7	52.5
Male	8163	28,140	29.0	13,273	32,481	40.9	41.0
Race							
Caucasian	17,275	55,358	31.2	28,354	61,805	45.9	47.1
Asian, Native Hawaiian, or Pacific Islander	194	828	23.4	484	1182	40.9	74.8
African American	188	696	27.0	323	865	37.3	38.1
American Indian or Alaska Native	105	442	23.8	196	574	34.1	43.3
Other	78	399	19.5	182	653	27.9	43.1
>1 race reported	62	277	22.4	155	419	37.0	65.2
Hispanic or Latino							
Yes	249	1113	22.4	400	1606	24.9	11.2
No	17,653	56,887	31.0	29,294	63,892	45.8	47.7
Education							
<HS	3134	10,975	28.6	4403	10,434	42.2	47.6
HS/GED	7286	23,622	30.8	11,828	26,001	45.5	47.7
Some college	3243	10,198	31.8	5407	12,149	44.5	39.9
Associate or Bachelor's degree	2873	9047	31.8	5443	11,441	47.6	49.7
Master's degree or higher	1150	3278	35.1	2255	4480	50.3	43.3
Unknown	216	880	24.5	358	993	36.1	47.3

GED, general educational development; HS, high school; POLST, Physician Orders for Life-Sustaining Treatment.

**Table 2.** Top 10 Natural Causes of Death with Proportion of Registered Physician Orders for Life-Sustaining Treatment Forms at Death and Proportional Change between Cohorts

	*Cohort 1: 2010–2011 decedents*				*Cohort 2: 2015–2016 decedents*				
*Cause of death*	*All natural deaths (*n*)*	*Registered POLST (*n*)*	*Registered POLST (%)*	*Median time POLST completed before death (rounded to weeks)*	*All natural deaths (*n*)*	*Registered POLST (*n*)*	*Registered POLST (%)*	*Median time POLST completed before death (rounded to weeks)*	*Difference% increase in registered POLST*
Malignant neoplasms	14,980	6146	41.0	4	16,198	7685	47.4	7	15.6
Heart diseases	11,799	2967	25.1	7	13,656	5689	41.7	30	66.1
Alzheimer's disease and dementia	6118	2122	34.7	9	6850	4246	62.0	52	78.7
Chronic lower respiratory diseases	3918	1211	30.9	7	4181	2015	48.2	28	56.0
Cerebrovascular diseases	3603	996	27.6	5	3203	1387	43.3	35	56.9
Diabetes mellitus	2126	526	24.7	6	2381	949	39.9	32	61.5
Liver disease	1024	246	24.0	3	1271	383	30.1	4	25.4
Influenza and pneumonia	779	162	20.8	6	892	346	38.8	49	86.5
Parkinson's disease	695	248	35.7	6	871	573	65.8	37	84.3
Nephritis	699	221	31.6	5	793	421	53.1	26	68.0
All others	12,259	3057	24.9	6	15,202	6000	39.5	31	58.6
All causes	58,000	17,902	30.9	5	65,498	29,694	45.3	21	46.6

Using proportions to account for the increase in deaths that were not evenly distributed, there was little or no increase in POLST use for decedents aged 18 or younger or 19–44 years old, the age groups with the fewest numbers of decedents (2.9% of all deaths). However, for decedents aged 45 and older, there was a steady rise in POLST use by age between the time periods: 18.6% for ages 45–64, 31.7% for ages 65–74, 43.7% for ages 75–84, 64.5% for ages 85–94, and 83.3% for age 95 and older (Table 1).

POLST use among women increased by 52.5% (from 32.6% to 49.7%), at a much higher rate than men, 41.0% (from 29% to 40.9%, *p* ≤ 0.001).

The majority of Oregon decedents are Caucasian and remain more likely than other racial/ethnic groups to use POLST. POLST use among Caucasians increased by 47.1% from Cohort 1 to Cohort 2. While they still do not use POLST as consistently as Caucasians, Asians and Pacific Islanders (74.8% increase) and those reporting more than one race (65.2% increase) began to close the gap. African Americans (38.1% increase), Native Americans, and Native Alaskans (43.3% increase), and particularly Hispanic/Latinos (11.2%), saw the gap widen.

There remains an education gap in POLST use with the least educated using POLST 42.2% in Cohort 2 and the most educated using POLST 50.3% of the time. However, in Cohort 2, all education categories increased at rates greater than the highest category (47.6% to 49.7% compared to 43.3%), so the gap closed slightly.

Malignant neoplasm remains the most common cause of death in Oregon and accounts for the largest number of POLST forms, but compared with Cohort 1 when malignant neoplasms were the cause of death most likely to have a POLST in the Registry, now Alzheimer's disease and dementia, chronic lower respiratory diseases, Parkinson's disease, and nephritis have a higher percentage. Looking at the amount of change from Cohort 1 to Cohort 2 by using the mean 46.6% increase in all causes of death for comparison, all causes of death (56.0% to 86.5%) except malignant neoplasms (15.6%) and liver disease (25.4%) increased at a rate higher than the mean.

The time between final POLST completion and death increased substantially from Cohort 1 to Cohort 2—a fourfold increase from 5 to 21 weeks. Alzheimer's disease/dementia and Parkinson's disease had an approximately sixfold increase in the median time between last POLST completion (from 9 to 52 weeks, and from 6 to 37 weeks, respectively). Median time from final POLST to death did not change substantially for decedents with causes of death of cancer or liver disease.

Orders for “CPR vs. DNR” (Section A) and “medical interventions” (Section B) were significantly different among time periods (Table 3). While orders for “DNR” and “comfort measures only” were still the most prevalent and showed the greatest increases in absolute numbers, orders for “attempt CPR,” “limited treatment,” and “full treatment” increased at proportionally greater rates.

**Table 3.** Registered Physician Orders for Life-Sustaining Treatment Form Orders with Absolute Difference between Cohorts

	*Cohort 1: 2010–2011 decedents*		*Cohort 2: 2015–2016 decedents*		
	n	*%*	n	*%*	*Cohort difference*^a^*% change in order selection*
Section A orders^*^					
CPR	1334	7.5	3810	12.8	185.6
DNR	16,568	92.5	25,875	87.1	56.2
Section B orders^*^					
Full treatment	1153	6.4	3359	11.3	191.3
Limited treatment	4787	26.7	10,839	36.5	126.4
Comfort measures only	11,836	66.1	15,316	51.6	29.4
Not filled out	126	0.7	180	0.6	42.9

^a^ Calculated as absolute number increase between cohorts with Cohort 1 as denominator.

^*^ *p* < 0.001.

CPR, cardiopulmonary resuscitation; DNR, do not resuscitate.

**FIG. 2.**
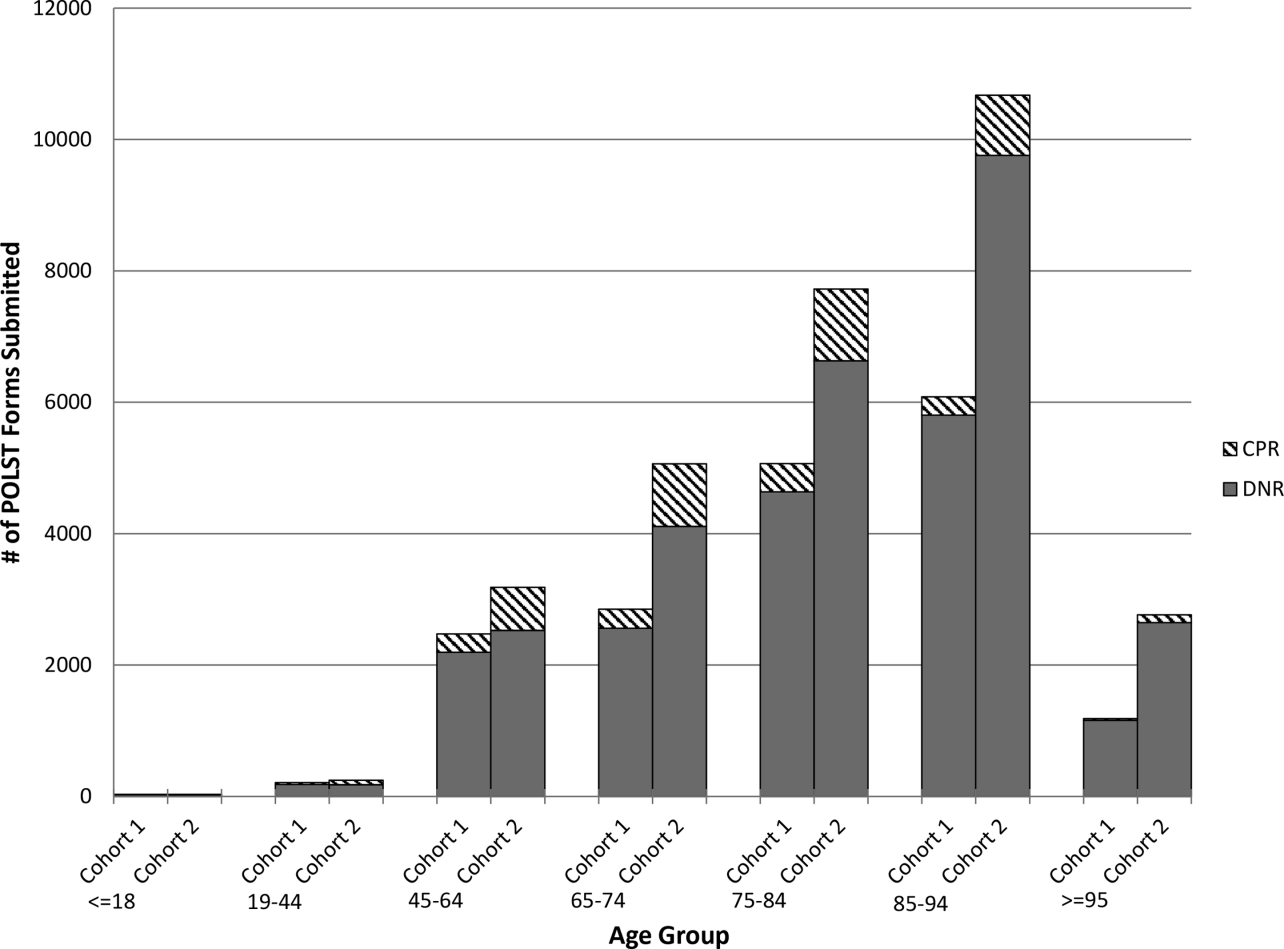
Number of registered POLST form orders for DNR and CPR by cohort and age group. CPR, cardiopulmonary resuscitation; DNR, do not resuscitate.

Figure 2 shows a notable increase in POLST use in those older than 85 years. The median age of decedents with orders for “CPR” is substantially younger (age 84 vs. age 76). Proportional increases in “attempt CPR” orders were seen in all age groups from Cohort 1 to Cohort 2, but as Figure 2 illustrates, the proportions got smaller with each successive age category.

In the logistic regression models, increased age, female sex, Caucasian non-Hispanic race, higher education, and death due to cancer or dementia were all associated with decreased likelihood of having orders to “attempt CPR” and/or for “full treatment” on the final POLST form before death. Controlling for all these variables, decedents in Cohort 2 still had an increased likelihood of having more aggressive orders: “attempt CPR” (odds ratio [OR] = 1.86, 95% confidence interval [CI] = 1.74–1.99) and “full treatment” (OR = 1.89, 95% CI = 1.76–2.03) (Table 4).

**Table 4.** Logistic Regression Results with Outcome of Cardiopulmonary Resuscitation Orders (Model 1) and Full Treatment Orders (Model 2)

	*Model 1: Outcome: CPR orders on final POLST form*	*Model 2: Outcome: full treatment orders on final POLST form*
	*odds ratio (95% CI)*	*odds ratio (95% CI)*
Cohort	1.864 (1.744–1.993)	1.889 (1.759–2.028)
Age	0.966 (0.964–0.968)	0.965 (0.962–0.967)
Female	0.843 (0.794–0.895)	0.832 (0.781–0.886)
>HS/GED	0.953 (0.898–1.012)	0.941 (0.883–1.003)
White	0.693 (0.611–0.786)	0.621 (0.547–0.706)
Cancer	0.418 (0.388–0.451)	0.399 (0.368–0.432)
Alzheimer's or dementia	0.464 (0.413–0.522)	0.408 (0.358–0.465)

CI, confidence interval.

## Discussion

While deaths in Oregon increased 12.9% between the cohorts, the rate of increase in decedents with a POLST in the Registry increased nearly four times that rate (46.6%). The change represents an absolute rise from 30.9% to 45.3% resulting in nearly half of those dying of natural causes having a POLST form in the Registry at the time of death in 2015–2016. It is not possible to distinguish from our data whether this increase is due to increased POLST use, or increased use of the POLST Registry, and probably both factors. For both POLST and Registry use, as with any innovation diffusion, there have been both early and late adopters and varying consistency among health systems, clinics, hospice programs, and skilled nursing facilities. There were substantially more senders in Cohort 2 (2015–2016), and form submissions per year continue to rise.^21^ Economic influences may also be contributing to higher POLST use in Cohort 2. In 2011, the Affordable Care Act expanded the Medicare annual wellness visit to include end-of-life care planning. In 2016, ACP conversations became a billable Medicare benefit.^22,23^ In addition, there is an overall reduction of transitions and expenditures near the end of life and a growing focus on coordinated care and ACP for patients with advanced illness.^24,25^

That said, there are several strong arguments for this increase being more related to registry use rather than the use of POLST alone. First, POLST has been used in Oregon since 1993 and use has been widespread for more than 20 years.^2^ Second, in Cohort 1, the Registry was brand new and while new POLST forms were required to be submitted, there was not a clear mandate for health systems to search through medical records to identify and submit older pre-existing POLST forms.^26^ Third, the substantial increases in time between last POLST form and death from Cohort 1 to Cohort 2 point to the Registry being brand new in Cohort 1 and relatively more “mature” in Cohort 2. While most of the decedents in Cohort 1 had their POLST forms completed during Cohort 1, decedents in Cohort 2 could have had their forms completed any time from 2010 to 2016. Maturity is relative because while Oregon's POLST Registry is the oldest and most established of its kind, it continues to evolve.^27^

Older adults remain the vast majority of POLST users. Although the median age of POLST users increased by only 1 year from age 82 in Cohort 1 to age 83 in Cohort 2, the oldest old had the greatest proportional increases in POLST use: 64.5% for ages 85–94 and 83.3% for ages 95+. With this shift toward the oldest old, POLST use increased at a faster rate among women than among men. We also see the greatest increase in POLST use among the causes of death diagnoses most commonly associated with the frailty trajectory—Alzheimer's disease and Parkinson's disease. Two-thirds of persons with these diagnoses now have a POLST form in the Registry at the time of death.

It is important to note that the median period of time between final POLST form completion and death for persons with Alzheimer's disease and other forms of dementia is 52 weeks. Many patients with Alzheimer's disease and other frailty diagnoses complete POLST forms substantially before the time of their death. This has policy implications because currently, POLST educational materials suggest using the surprise question (“Would you be surprised if the patient died in the next year?”) to determine who should have the opportunity to complete a form. The surprise question may be helpful for patients with other illnesses such as cancer, but it may be an especially poor fit for those with a much slower decline with advanced age, cognitive impairment, and frailty.^10,28–32^ In a recent study about ACP in those with documentation of frailty, only 10% died in a 12-month period and yet many wanted to engage in ACP and have a DNR order.^33^

Compared with Cohort 1, Cohort 2 contains disproportionate increases in all “higher” levels of life-sustaining treatment orders, a finding that was confirmed for “attempt CPR” and “full treatment” orders in the logistic regression models. Figure 2 shows the proportions of “attempt CPR” versus “DNR” by age category to demonstrate two important points—first, while the proportion of patients opting to “attempt CPR” increased in all age groups, the inverse relationship between “attempt CPR” and increasing age remained in effect with a lower percentage of “attempt CPR” orders with each increasing age category above 45 years. Second, while the proportion of “attempt CPR” and “full treatment” orders increased dramatically, the absolute numbers of new “DNR” and “CMO” orders were substantially greater.

It is worth considering what this shift toward more aggressive treatment represents. It may be an artifact caused by hospice programs that routinely use POLST, but do not routinely submit their forms to the Registry, telling patients to call hospice rather than Emergency Medical Services (EMS). Thus, the Registry would not reflect the most current POLST form for those decedents. It may also be that POLST use is spreading to patients who previously would not have completed POLST and who also have preferences for more aggressive treatment. If these orders for aggressive treatment accurately reflect the preferences of these decedents, that would not be a cause for concern, however, there is another possibility related to the increases in time between POLST completion and death. Previous studies of changes in POLST forms over time show that most patients who complete more than one form move toward lower levels of treatment as they approach death.^6,15,17–19^ Therefore, it seems plausible that the increasing proportions of patients with orders for higher levels of treatment could be related to the increasing time between POLST completion and death. This begs the following question: how often do POLST orders for “attempt CPR” and “full treatment” completed months before death still reflect patient preferences at the time of death?

While the secular trend toward more and earlier ACP and serious illness communication is extremely positive,^34–40^ documenting patient preferences to “attempt CPR” and “full treatment” years before death may have unintended consequences if the orders are not revisited when health status changes.^41^

This study has a number of limitations. Despite being the most widely used POLST Registry in the United States,^27^ the Registry still underestimates POLST use in Oregon. Some health care organizations fail to submit forms consistently, and some patients opt out of having their forms registered. A large number of submitted POLST forms lack legible critical information (such as patient identifiers, signatures, or dates) and cannot be entered into the Registry.^21^ We used death certificate data to classify cause of death. For some diseases, such as end-stage dementia, substantial underreporting occurs. In one prospective study of nursing home residents with end-stage dementia, pneumonia was reported more frequently as the cause of death than dementia.^42^

Finally, the study uses data from only one state, Oregon, and its unique POLST program.^43^ End-of-life practices vary by region and so our data, particularly life-sustaining treatment orders, may not reflect other states, especially those with greater racial and ethnic diversity.^44,45^

## Conclusion

Based on these findings, we recommend a different approach to the timing of POLST completion for those with declining cognitive function and frailty, acknowledging that significantly more than a year before death they may wish to set limits on some medical treatments. We also assume that, as long as the presumption for those without POLST remains firmly to “attempt CPR” and provide “full treatment,” there is a greater potential for harm in documenting these preferences in patients who are not terminally ill and may have years left to live. As an alternative to the question “Would you be surprised if this person were to die in the next year?” we propose testing the following criteria for POLST in frail elders. (1) Patient has had serious illness conversations, including values, priorities, and preferences for life-sustaining treatment. (2) They are beginning to need assistance because of physical or cognitive decline. (3) Patient expresses an interest in setting some limits on life-sustaining treatment.
